# Supplementary material for: The interactome of intact mitochondria by cross-linking mass spectrometry provides evidence for coexisting respiratory supercomplexes
Source: Mol Cell Proteomics. 2017 Dec 8;17(2):216–32. doi: 10.1074/mcp.RA117.000470 (PMC5795388; doi:10.1074/mcp.RA117.000470)
Supplement: Supplemental Data [file supp_RA117.000470_133922_0_supp_23978_fzffwf.zip › spectra_annotation/mito_spectra_annotation/444-1-1-1-3-1.pdf]

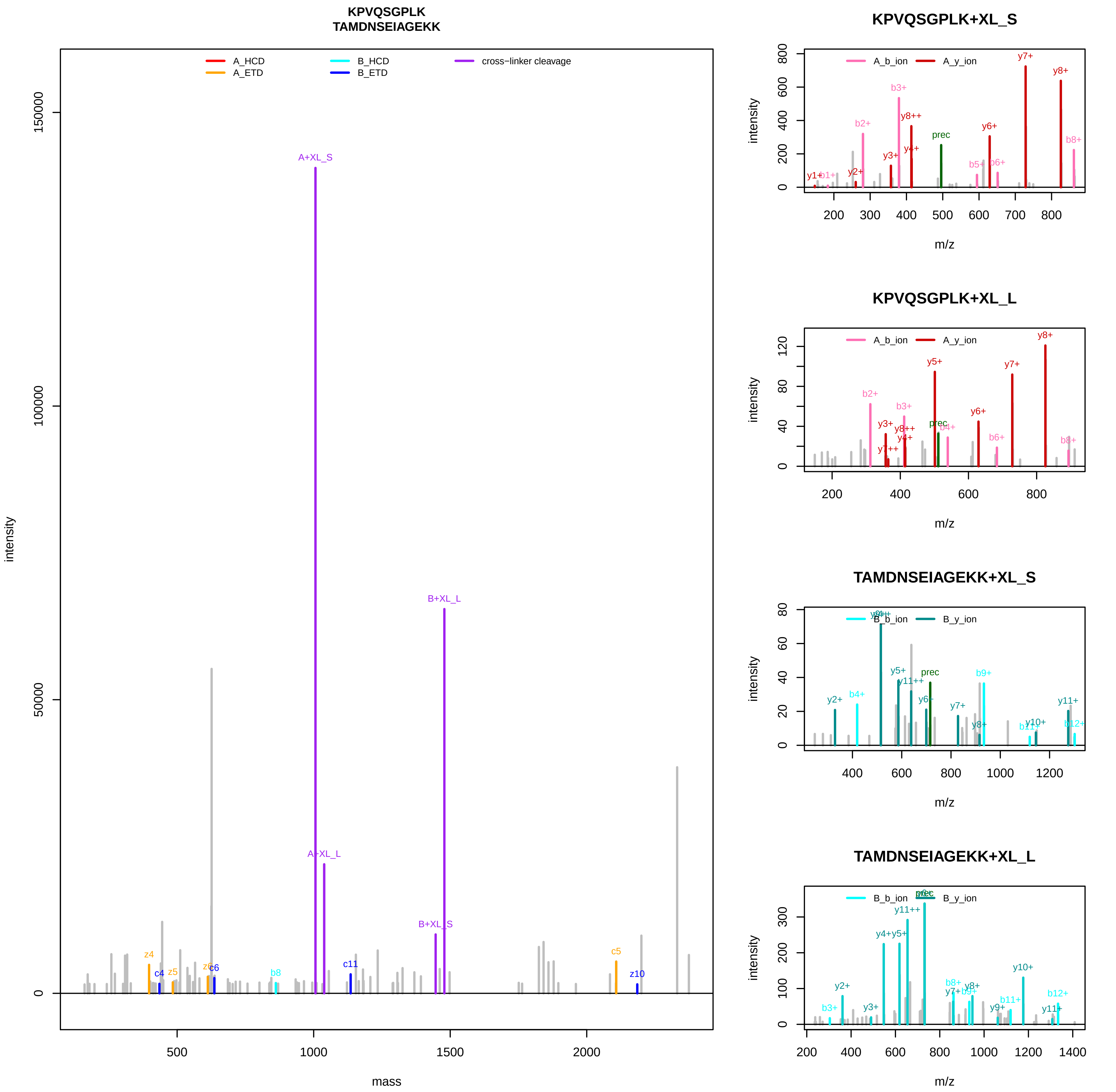

KPVQSGPLK
TAMDNSEIAGEKK
A_HCD
A_ETD
B_HCD
B_ETD
cross−linker cleavage
A+XL_S
B+XL_L
A+XL_L
B+XL_S
z4
c4
z5
c6
b8
c11
c5
z10
intensity
mass
0
50000
100000
150000
500
1000
1500
2000
KPVQSGPLK+XL_S
A_b_ion
A_y_ion
y1+
b1+
y2+
b2+
y3+
b3+
y8++
y4+
prec
b5+
b6+
y6+
y7+
y8+
b8+
m/z
KPVQSGPLK+XL_L
y5+
y7++
b4+
TAMDNSEIAGEKK+XL_S
B_b_ion
B_y_ion
y2+
b4+
y5+
y11++
y6+
y7+
y8+
b9+
b11+
y10+
y11+
b12+
TAMDNSEIAGEKK+XL_L
b3+
y3+
y4+y5+
y11++
b8+
y7+
b9+
y8+
y9+
b11+
y10+
y11+
b12+
